# Incidence of and risk factors for post-parathyroidectomy hungry bone syndrome in patients with secondary hyperparathyroidism

**DOI:** 10.1080/0886022X.2020.1841655

**Published:** 2020-11-03

**Authors:** Kittrawee Kritmetapak, Sawinee Kongpetch, Wijittra Chotmongkol, Yutapong Raruenrom, Sakkarn Sangkhamanon, Chatlert Pongchaiyakul

**Affiliations:** a Division of Nephrology, Department of Medicine, Faculty of Medicine, Khon Kaen University, Khon Kaen, Thailand; b Kidney Center of Excellence, Srinagarind Hospital, Khon Kaen University, Khon Kaen, Thailand; c Division of Nuclear Medicine, Department of Radiology, Faculty of Medicine, Khon Kaen University, Khon Kaen, Thailand; d Department of Pathology, Faculty of Medicine, Khon Kaen University, Khon Kaen, Thailand; e Division of Endocrinology and Metabolism, Department of Medicine, Faculty of Medicine, Khon Kaen University, Khon Kaen, Thailand

**Keywords:** Dialysis, hungry bone syndrome, hyperparathyroidism, hypocalcemia, parathyroidectomy

## Abstract

**Background:**

Hungry bone syndrome (HBS) following parathyroidectomy is associated with severe hypocalcemia and increased morbidity. This study aims to determine the incidence and risk factors of post-parathyroidectomy HBS in dialysis patients with secondary hyperparathyroidism (SHPT).

**Methods:**

A retrospective cohort study was conducted, and medical records of patients with SHPT requiring parathyroidectomy between January 2014 and January 2020 were reviewed. HBS was defined as the requirement of intravenous calcium administration due to hypocalcemia-related symptoms and/or reductions in serum calcium concentration (<8.4 mg/dL) within 72 h after parathyroidectomy.

**Results:**

A total of 130 dialysis patients were enrolled. The majority of patients (85.4%) received hemodialysis and the remaining patients (14.6%) received peritoneal dialysis. Ectopic parathyroid glands were identified in 6.6% of patients by preoperative parathyroid scintigraphy. Diffuse parathyroid hyperplasia was the most common histopathological characteristic of SHPT (90.8%). HBS occurred in 82.3% of patients following parathyroidectomy. Preoperative serum intact parathyroid hormone (iPTH) concentration was significantly correlated with serum calcium (*r* = −0.48, *p* < 0.01) and alkaline phosphatase (ALP) concentration (*r* = 0.71, *p* < 0.01). Patients with HBS had significantly longer hospital stays than patients without (8 versus 3 days, *p* < 0.01). Based on multiple logistic regression analysis, young age (≤45 years), high preoperative serum ALP (>420 IU/L) and iPTH (>1,000 pg/mL), and absence of preoperative hypercalcemia (>10.2 mg/dL) were significantly associated with HBS.

**Conclusions:**

Post-parathyroidectomy HBS is common in dialysis patients with SHPT. Young age, high preoperative serum ALP and iPTH, and low preoperative serum calcium concentrations were important risk factors for HBS.

## Introduction

Secondary hyperparathyroidism (SHPT) encompasses the biochemical abnormalities of chronic kidney disease-mineral and bone disorder (CKD-MBD) and develops in the majority of patients with end-stage renal disease (ESRD) who require dialysis [1]. Chronic hyperphosphatemia, hypocalcemia, and 1,25-dihydroxyvitamin D [1,25(OH)_2_D] deficiency are the primary factors that stimulate parathyroid hormone (PTH) synthesis and parathyroid cell proliferation, resulting in the autonomous hypersecretion of PTH and the development of hyperplastic parathyroid glands [2]. Patients with SHPT refractory to pharmacological therapy, including calcimimetics and active vitamin D analogues, require surgical parathyroidectomy [3,4]. Subtotal parathyroidectomy or total parathyroidectomy with or without parathyroid autotransplantation is indicated in these cases. It is estimated that approximately 15% and 38% of patients require parathyroidectomy after 10 and 20 years, respectively, of ongoing dialysis treatment [5]. Successful parathyroidectomy results in a dramatic reduction in serum PTH concentrations and improvement of clinical symptoms such as bone pain and pruritus. Moreover, this procedure also improves patient survival [6–9], decreases resistance to erythropoietin, increases bone mineral density, and reduces risk of fractures [10] in dialysis patients with SHPT.

Hungry bone syndrome (HBS), characterized by rapid, profound, and prolonged hypocalcemia, is a notable potential complication after parathyroidectomy. Patients with HBS usually have a severe reduction in total serum calcium concentration to less than 8.4 mg/dL and/or prolonged hypocalcemia for more than 4 days postoperatively [11]. The abrupt reduction in serum PTH concentration after parathyroidectomy is believed to modify the balance between bone formation and resorption, favoring bone formation with greatly increased skeletal uptake of calcium, which results in severe hypocalcemia. Although this is generally a transient phenomenon that resolves with calcium and active vitamin D supplementation, patients with HBS can develop severe hypocalcemia with life-threatening complications, i.e. cardiac arrhythmia, tetany, and laryngeal stridor. Previous studies have reported that the incidence of HBS varies from 28% to 88% in SHPT patients undergoing parathyroidectomy [12–16], depending on the criteria for the diagnosis of HBS. Furthermore, the risk factors for HBS in patients with SHPT remain controversial [12–18].

Despite current advances in our understanding of the pathophysiology of SHPT, there have been few studies on post-parathyroidectomy HBS in patients with SHPT. To prevent and manage this postoperative complication, early identification of patients at high risk of developing HBS is crucial. Therefore, this study aims to determine the incidence of HBS and examine its associated risk factors in dialysis patients who underwent parathyroidectomy for SHPT.

## Materials and methods

### Subjects and setting

The clinical, biochemical, and preoperative parathyroid scintigraphy data of dialysis patients with SHPT who underwent parathyroidectomy in a tertiary setting (Srinagarind Hospital, Thailand) from January 2014 through January 2020 were reviewed and analyzed retrospectively. Data were collected from histopathological reports of resected parathyroid tissues, requisition forms, and discharge summaries. Non-dialysis CKD patients, kidney transplant patients, and patients with inadequate clinical data were excluded. The study was performed in accordance with the principles of the Declaration of Helsinki and was approved by Khon Kaen University Faculty of Medicine Ethics Committee (IRB00001189).

### Laboratory tests and parathyroid scintigraphy

Laboratory parameters, including hemoglobin, albumin-corrected serum calcium, phosphate, alkaline phosphatase (ALP), total 25-hydroxyvitamin D [25(OH)D], and intact parathyroid hormone (iPTH) concentration, were retrieved from electronic medical records and evaluated. Serum iPTH concentrations were measured using an iPTH immunoassay and a Roche Cobas e411 analyzer. Preoperative parathyroid localization was performed using dual tracer, dual phase, or combined dual tracer dual phase protocol. Parathyroid planar imaging was conducted using a Vertex V60 EPIC HP dual-headed gamma camera (ADAC, CA, USA), Genesys single-headed gamma camera (ADAC, CA, USA), or Discovery NM/CT 670 (GE Healthcare, IL, USA) equipped with a low energy high-resolution collimator with a 128 × 128 imaging matrix and an energy peak set at 140 keV ± 10%. In addition to planar imaging, concurrent single photon emission computed tomography-computerized tomography (SPECT/CT) imaging was performed in most patients at the discretion of the nuclear medicine physician.

### Indication for parathyroidectomy and term definition

The indications for parathyroidectomy in this cohort included (1) persistently elevated serum iPTH levels >800 pg/mL complicated with hyperparathyroid-related clinical symptoms (bone pain, fracture, refractory pruritus), refractory hypercalcemia, hyperphosphatemia, or calciphylaxis or (2) serum iPTH levels >1000 pg/mL (>6 months) in asymptomatic patients that is refractory to medical treatment including active vitamin D analogues and calcimimetics [5,19]. Subtotal parathyroidectomy or total parathyroidectomy with or without parathyroid autotransplantation was performed at the discretion of the operating surgeon. A reduction in serum iPTH concentration of more than 70% compared with preoperative baseline values following the removal of hyperfunctioning parathyroid glands was considered to indicate successful parathyroidectomy in patients with SHPT [20].

All resected parathyroid gland specimens were reviewed by an experienced pathologist to identify the presence of parathyroid hyperplasia or adenoma. Parathyroid hyperplasia was defined as an adaptive increase in parathyroid parenchymal mass resulting from proliferation of chief, oxyphil, and transitional cells in multiple parathyroid glands. Parathyroid adenoma was defined as encapsulated monoclonal cell proliferation composed predominantly of chief cells and involving a single parathyroid gland. HBS was defined as the requirement of intravenous calcium administration due to clinical symptoms of hypocalcemia (e.g. tingling, muscle spasms, tetany) and/or a rapid postoperative reduction in serum calcium concentration to less than 8.4 mg/dL during the first 72 h after parathyroidectomy, despite optimization of supportive therapy including oral calcium and vitamin D supplementation [21–23].

### Statistical analysis

Statistical analysis included computing (i) the frequency counts and percentages for the categorical variables and (ii) the means ± standard deviations (SD) or medians with interquartile ranges (IQ25 and IQ75) for the continuous variables. Comparisons of categorical variables between patients with and without HBS were made using the chi-squared or Fisher’s exact test, as appropriate. Continuous variables were tested for normality using a Shapiro-Wilk test and were compared using Student’s t-test or the Kruskal-Wallis test. Median with interquartile range was used where distribution was not normal. The correlation between preoperative serum calcium, ALP, and iPTH was analyzed using Spearman’s correlation test. Univariate and multivariable logistic regression analyses were used to determine the associations between potential risk factors and HBS. *p*-values <0.05 were considered statistically significant. All statistical analysis was performed using STATA version 14.0.

## Results

**Figure 1.**
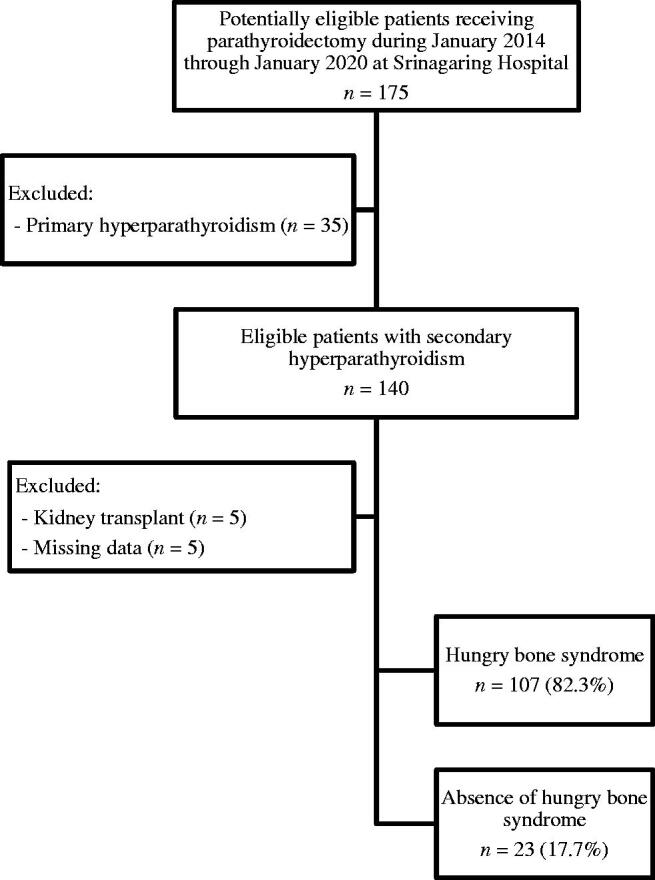
Subject disposition for the study cohort.

A total of 130 dialysis patients with SHPT who underwent parathyroidectomy were recruited (Figure 1). The demographic and clinical characteristics of the patients are shown in Table 1. Sixty-nine patients (53.1%) were female, with a mean age of 44.5 ± 11.7 years. Most patients (85.4%) received chronic hemodialysis, and the mean duration of dialysis was 7.7 ± 3.9 years. Preoperative serum iPTH concentration was negatively correlated with serum calcium (*r* = −0.48, *p* < 0.01) and positively correlated with serum ALP (*r* = 0.71, *p* < 0.01). Preoperative parathyroid scintigraphy with SPECT/CT detected multi-gland hyperplasia in most patients. Ectopic parathyroid glands were identified in 6.6% of patients undergoing preoperative parathyroid scintigraphy and were predominantly located in the mediastinum. The median time from parathyroid scintigraphy to parathyroidectomy was 105 days (50–210). The most common surgical procedure was subtotal parathyroidectomy (62.3%). The majority of patients (90.8%) had parathyroid hyperplasia on histopathological diagnosis and the remaining patients (9.2%) had parathyroid adenoma.

**Table 1.** Demographic and clinical characteristics of the study participants at the time of parathyroidectomy.

Characteristics	All patients (*n* = 130)
Female sex, *n* (%)	69 (53.1%)
Age at time of surgery (years), mean ± SD	44.5 ± 11.7
Mode of renal replacement therapy, *n* (%)	
Hemodialysis	111 (85.4%)
Peritoneal dialysis	19 (14.6%)
Duration of dialysis (years), mean ± SD	7.7 ± 3.9
Underlying renal disease, *n* (%)	
Diabetes mellitus	8 (6.2%)
Hypertension	14 (10.8%)
Obstructive uropathy	16 (12.3%)
Chronic glomerulonephritis	19 (14.6%)
Renal cystic disease	4 (3.1%)
Drug-induced renal disease	2 (1.5%)
Congenital renal disease	2 (1.5%)
Unknown cause	65 (50.0%)
Symptoms, *n* (%)	
Bone pain	66 (50.8%)
Fracture	10 (7.7%)
Preoperative laboratory testing	
Corrected calcium (mg/dL), mean ± SD	10.0 ± 1.2
Phosphate (mg/dL), mean ± SD	5.1 ± 1.7
Alkaline phosphatase (IU/L), median (IQ25-75)	367 (199–743)
iPTH (pg/mL), median (IQ25-75)	2237 (1779–3038)
Ectopic location of parathyroid gland, *n* (%)	
Thyroid gland	1 (0.8%)
Mediastinum	7 (5.8%)
No ectopic parathyroid gland	113 (93.4%)
Histopathological diagnosis, *n* (%)	
Parathyroid hyperplasia	118 (90.8%)
Parathyroid adenoma	12 (9.2%)

iPTH: intact parathyroid hormone.

The incidence of HBS following parathyroidectomy in this study was 82.3%. The clinical data of patients with and without HBS are shown in Table 2. Among patients with HBS, 54.2% had serum calcium concentrations ≤7.5 mg/dL prior to intravenous calcium administration. Those with HBS were younger on average than those without and a higher percentage were females. Median hospital stay was also significantly longer in HBS patients (8 versus 3 days, *p* < 0.01). Regarding preoperative laboratory testing, mean serum calcium concentration was significantly lower but median serum ALP and iPTH concentrations were significantly higher in patients with HBS than in those without. The prevalence of preoperative hypercalcemia (>10.2 mg/dL) was significantly lower in patients with HBS (31.8 versus 60.9%, *p* < 0.01), and those with marked elevations in serum ALP (greater than three times the upper limits of normal; >420 IU/L) and iPTH (>1000 pg/mL) were significantly higher (60.7% and 98.1%, respectively, in HBS patients versus 47.8% and 82.6%, respectively, in non-HBS patients). Neither duration of dialysis, the presence of bone pain or fracture, previous parathyroidectomy, type of parathyroid surgery, preoperative serum phosphate, 25(OH)D, nor hemoglobin concentrations appeared to differ between the two groups. Preoperative oral elemental calcium and active vitamin D analogue (alfacalcidol, calcitriol) dosages were also comparable between groups.

**Table 2.** Comparison of clinical data of patients with and without hungry bone syndrome.

Characteristics	Hungry bone syndrome		*p* Value
	Yes (*n* = 107)	No (*n* = 23)	
Female sex, *n* (%)	60 (56.1%)	9 (39.1%)	0.04
Age at time of surgery (years), mean ± SD	42.6 ± 11.9	49.5 ± 10.1	0.03
Age ≤ 45 years, *n* (%)	65 (60.7%)	7 (30.4%)	<0.01
Mode of renal replacement therapy, *n* (%)			
Hemodialysis	93 (86.9%)	18 (78.3%)	<0.01
Peritoneal dialysis	14 (13.1%)	5 (21.7%)	
Duration of dialysis (years), median (IQ25-75)	7.5 (5–10)	8.7 (5–12)	0.08
Presence of diabetes, *n* (%)	8 (7.5%)	0 (0%)	0.18
Preoperative symptoms, *n* (%)			
Bone pain	50 (46.7%)	11 (47.8%)	0.95
Fracture	9 (8.4%)	1 (4.3%)	0.51
Previous parathyroidectomy, *n* (%)	12 (11.2%)	5 (21.7%)	0.17
Current surgical procedure, *n* (%)			
Subtotal parathyroidectomy	66 (61.7%)	15 (65.2%)	0.38
Total parathyroidectomy with parathyroid autotransplantation	21 (19.6%)	5 (21.7%)	0.26
Total parathyroidectomy without parathyroid autotransplantation	20 (18.7%)	3 (13.1%)	0.17
Preoperative medications			
Elemental calcium (mg/day), median (IQ25-75)	400 (0–1,200)	0 (0–750)	0.19
Use of non-calcium-based phosphate binder, *n* (%)	29 (27.1%)	9 (39.1%)	0.25
Use of alfacalcidol or calcitriol, *n* (%)	52 (48.6%)	16 (69.6%)	0.05
Alfacalcidol (mcg/week), median (IQ25-75)	4.5 (2.25–9.00) *n* = 31	6 (6–8) = 11	0.18
Calcitriol (mcg/week), median (IQ25-75)	3 (1.5–6.0) *n* = 21	3.75 (2.0–4.5) *n* = 5	0.63
Preoperative laboratory testing			
Corrected calcium (mg/dL), mean ± SD	9.4 ± 1.1	10.4 ± 1.5	0.04
Presence of hypercalcemia (>10.2 mg/dL), *n* (%)	34 (31.8%)	14 (60.9%)	<0.01
Phosphate (mg/dL), mean ± SD	5.2 ± 1.7	4.7 ± 1.8	0.91
Presence of hyperphosphatemia (>5.5 mg/dL), *n* (%)	44 (41.1%)	8 (34.8%)	0.58
Alkaline phosphatase (IU/L), median (IQ25-75)	402 (217–728)	179 (76–541)	0.03
Presence of elevated alkaline phosphatase (>420 IU/L), *n* (%)	65 (60.7%)	11 (47.8%)	0.04
iPTH (pg/mL), median (IQ25-75)	2327 (1816–3,240)	1645 (1239–2050)	<0.01
Presence of elevated iPTH (>1,000 pg/mL), *n* (%)	105 (98.1%)	19 (82.6%)	<0.01
25(OH)D (ng/mL), mean ± SD	37.1 ± 16.6	32.2 ± 11.8	0.19
Hemoglobin (g/dL), mean ± SD	9.9 ± 1.8	9.9 ± 2.1	0.50
Postoperative laboratory testing			
Corrected calcium (mg/dL) POD1, mean ± SD	8.6 ± 1.0	9.7 ± 1.0	<0.01
Corrected calcium (mg/dL) prior to intravenous calcium administration, mean ± SD	7.4 ± 0.7	–	–
Phosphate (mg/dL) POD1, mean ± SD	4.8 ± 1.8	4.9 ± 1.6	0.45
iPTH (pg/mL) POD1, median (IQ25-75)	152 (82–291)	366 (99–788)	<0.01
Percent reduction in iPTH POD1, median (IQ25-75)	94.2 (87.2–96.7)	70.3 (50.4–88.9)	<0.01
iPTH (pg/mL) POM3, median (IQ25-75)	52 (19–303)	364 (129–969)	<0.01
Percent reduction in iPTH POM3, median (IQ25-75)	89.2 (77.0–99.2)	55.9 (30.0–86.5)	<0.01
Duration of hospital admission (days), median (IQ25-75)	8 (5–11)	3 (2–6)	<0.01
Discharge medication			
Elemental calcium (mg/day), median (IQ25-75)	3200 (2400–4800)	1200 (400–2400)	<0.01
Alfacalcidol (mcg/day), median (IQ25-75)	1 (1–2) *n* = 75	0.5 (0.25–1) *n* = 12	<0.01
Calcitriol (mcg/day), median (IQ25-75)	2 (0.5–2) *n* = 32	0.5 (0.5–1) *n* = 8	<0.01

iPTH: intact parathyroid hormone; POD: postoperative day; POM: postoperative month.

Median serum iPTH concentrations among patients with HBS had decreased to 152 pg/mL (82–291) at postoperative day 1 and to 52 pg/mL (19–303) at postoperative month 3, representing median reductions of 94.2% (87.2–96.7) and 89.2% (77.0–99.2), respectively (*p* < 0.01). Those among patients without HBS had decreased to 366 pg/mL (99–788) at postoperative day 1 and to 364 pg/mL (129–969) at postoperative month 3, representing median reductions of 70.3% (50.4–88.9) and 55.9% (30.0–86.5), respectively (*p* = 0.03). Overall, reductions in iPTH post-operation were more significant in HBS patients than in those without HBS (*p* < 0.01). In addition, patients with HBS were discharged on significantly higher dosages of oral elemental calcium and active vitamin D analogues.

According to univariate analysis, female sex, younger age, lower preoperative serum calcium concentration, higher preoperative serum ALP and iPTH concentrations, and larger decreases in post-parathyroidectomy iPTH concentration were significantly associated with HBS. However, only age ≤45 years [odds ratio (OR) 2.67, 95% confidence interval (CI) 1.96–7.48, *p* = 0.01], preoperative serum ALP >420 IU/L (OR 1.14, 95% CI 1.01–1.37, *p* = 0.03), and preoperative serum iPTH >1000 pg/mL (OR 4.61, 95% CI 1.24–23.48, *p* = 0.04) were significantly associated with HBS by multivariate logistic regression analysis (Table 3). Moreover, preoperative hypercalcemia (>10.2 mg/dL) was significantly inversely associated with HBS (OR 0.37, 95% CI 0.14–0.62, *p* = 0.03).

**Table 3.** Multiple logistic regression model of risk factors for hungry bone syndrome.

Variable	Odds ratio	95% Confidence interval	*p* Value
Age ≤45 years	2.67	1.96–7.48	0.01
Preoperative serum corrected calcium >10.2 mg/dL	0.37	0.14–0.62	0.03
Preoperative serum alkaline phosphatase >420 IU/L	1.14	1.01–1.37	0.03
Preoperative serum iPTH >1000 pg/mL	4.61	1.24–23.48	0.04

## Discussion

The incidence of post-parathyroidectomy HBS is high in dialysis patients with SHPT. We found that young age (≤45 years), high preoperative serum ALP (>420 IU/L) and iPTH (>1000 pg/mL), and absence of preoperative hypercalcemia (>10.2 mg/dL) were important risk factors for HBS. Further, our study adds to the literature on the absence of effect of type of parathyroidectomy (subtotal parathyroidectomy or total parathyroidectomy with or without parathyroid autotransplantation) and dosage of active vitamin D analogues (alfacalcidol, calcitriol) on the HBS occurrence.

The international guidelines established by the Kidney Disease: Improving Global Outcomes (KDIGO) organization recommend that ESRD patients with severe SHPT who are refractory to medical therapy, including calcimimetics and active vitamin D analogues, undergo parathyroidectomy [4]. Although parathyroidectomy has been shown to improve renal osteodystrophy, reduce fracture risk, and decrease mortality in patients with SHPT, HBS remains a clinically significant complication following parathyroidectomy [24]. HBS is characterized by a rapid, profound, and persistent hypocalcemia associated with hypophosphatemia, hypomagnesemia, and is exacerbated by an abrupt reduction in serum PTH concentrations, which follows parathyroidectomy in patients with severe hyperparathyroidism and preoperative high bone turnover. Hypocalcemia-related symptoms range from mild (including tingling and muscle cramps) to severe (seizure, cardiac arrhythmia, and overt heart failure, especially in ESRD patients with latent myocardial dysfunction) [25,26]. Therefore, accurate and early identification of SHPT patients at high risk of developing HBS could potentially reduce postoperative complications.

The lack of well-defined criteria for the diagnosis of HBS makes it difficult to determine its true incidence. A systematic review of patients with primary hyperparathyroidism (PHPT) found that it ranged from 13% to 87% [21]. Likewise, the incidence of HBS in patients with SHPT varies widely, from 28% to 88% [12–16,27]. We defined HBS as the requirement of intravenous calcium administration due to hypocalcemia-related symptoms and/or a rapid postoperative reduction in serum calcium concentration (<8.4 mg/dL) and found an incidence of 82.3% in SHPT patients, which was comparable with previous studies [14,15]. Some studies have defined HBS based solely on serum calcium concentration immediately after parathyroidectomy. It is important to note, however, that some patients with HBS experience large reductions in postoperative serum calcium concentrations from baseline and might develop clinical symptoms, despite these concentrations still being within the normal range. Hence, the latter definition of HBS may have limited validity and lead researchers to underestimate its true incidence.

The findings in this study support those in the majority of previous reports that younger patients with SHPT are at increased risk for HBS [12,16,18,28]. Conversely, in patients with PHPT, advanced age was found to be associated with HBS [23,29,30]. Poor nutritional status, vitamin D deficiency, and diminished 1-alpha-hydroxylase activity in older patients with PHPT might confer a higher risk of developing HBS, whereas the aforementioned factors were equally present in ESRD patients with SHPT. It could be that increases in osteoblast-mediated bone formation following parathyroidectomy are more pronounced in younger than in older patients with SHPT, resulting in the aggravation of HBS.

The present study demonstrated that preoperative iPTH concentrations and the magnitude of postoperative iPTH reduction in patients with HBS were higher than patients without HBS. Moreover, a marked elevation in preoperative serum iPTH concentrations (>1000 pg/mL) had a high likelihood of predicting HBS. The incidence of HBS in our cohort was high, probably because of high preoperative serum iPTH concentrations (median, 2237 pg/mL) and a large percentage reduction in immediate postoperative iPTH concentrations (median, 92.5%). This finding might be explained by a net calcium translocation from the extracellular fluid into bone due to a precipitous fall in PTH concentration after a prolonged period of PTH exposure, which has been confirmed by a previous *in vitro* study [31]. Furthermore, this finding supports a previous hypothesis that a sudden decrease in serum PTH concentrations after sustained elevation following parathyroidectomy results in the unopposed action of osteoblasts and massive influx of calcium into bone, thereby causing severe hypocalcemia. Although hypercalcemia (>10.2 mg/dL) increases the risk of developing an arrhythmia, vascular calcification, and calciphylaxis [32,33], we found that it conferred a lower risk of HBS in patients with SHPT. Since the definition of HBS is based on low serum calcium concentrations and/or hypocalcemia-related symptoms, we speculate that patients with preoperative hypercalcemia might have a higher threshold for developing HBS.

Consistent with previous reports [12,15], our study revealed that preoperative serum ALP concentrations of more than three times the upper limit of normal (>420 IU/L) predicted the development of HBS among patients with SHPT. Preoperative serum ALP concentrations may also predict the degree and duration of hypocalcemia following parathyroidectomy [17,28,34]. Activity of ALP is important for the appropriate mineralization of bone. Osteoblasts normally express large amounts of ALP bone isoenzyme, and serum concentrations are typically elevated when osteoblast activity and bone formation rates are increased. In patients with SHPT, osteoblasts activated by elevated iPTH concentrations will further activate osteoclasts and lead to increased bone resorption. Therefore, preoperative serum ALP concentrations generally reflect the extent of bone histologic change in patients with high-turnover lesions of renal osteodystrophy [35], and values frequently correlate with serum PTH concentrations, as shown in our study. Nonetheless, some patients with long-standing SHPT still have trivial or no elevation in serum ALP concentrations, thus suggesting an extensive PTH-induced bone marrow fibrosis that is biochemically quiescent [36].

We explored the impact of preoperative serum 25(OH)D concentrations on the development of HBS. No significant differences in preoperative serum 25(OH)D concentrations were observed between patients with and without HBS. Notwithstanding, for patients who received active vitamin D analogues (alfacalcidol, calcitriol) preoperatively, the incidence of HBS following parathyroidectomy tended to be lower than in those who did not, but this difference did not reach statistical significance (48.6% versus 69.6%, *p* = 0.05). As patients reach ESRD, serum FGF23 and phosphate concentrations increase, whereas serum 1,25(OH)_2_D concentration decreases significantly. Impaired 1,25(OH)_2_D production is attributable to decreased serum 25(OH)D substrate concentration and diminished 1-alpha-hydroxylase activity caused by reduced renal mass [37]. In addition, FGF23 and phosphate also inhibit renal production of 1,25(OH)_2_D by suppressing 1-alpha-hydroxylase activity and simultaneously inducing 24-hydroxylase expression and inactive metabolite 24,25-dihydroxyvitamin D production [38]. Moreover, administration of 1,25(OH)_2_D induces 24-hydroxylase expression [39] and stimulates FGF23 secretion from osteocytes and osteoblasts [40], thereby potentially lowering 1,25(OH)_2_D concentrations in target tissues and mitigating the beneficial effects of active vitamin D analogues. We also could not exclude the possibility of uremic bone cells being associated with vitamin D receptor down-regulation and vitamin D resistance in the same manner as parathyroid cells [41]. Therefore, the exact role of active vitamin D analogues in the prevention of HBS in SHPT patients remains unknown.

Interestingly, results from patients with PHPT and parathyroid adenoma showed that increased parathyroid gland weight was a risk factor for HBS [23]. Nevertheless, no studies have shown parathyroid gland weight to be an HBS risk factor in patients with SHPT, partly due to different types of parathyroidectomy being conducted across studies. Parathyroid adenoma is the most common causes of PHPT, accounting for 80–85% of cases [42,43], whereas parathyroid hyperplasia is the primary histological finding in more than 90% in patients with SHPT, as shown in our study and various others [16,44–46]. The state of prolonged stimulation of parathyroid cell growth in ESRD patients due to chronic hyperphosphatemia, hypocalcemia, and 1,25(OH)_2_D deficiency results in diffuse parathyroid hyperplasia. Another important pathogenic factor in some patients with severe SHPT is neoplastic transformation of preexisting polyclonal parathyroid hyperplasia, leading to autonomous overgrowth by a monoclonal parathyroid neoplasia [47,48]. The underlying mechanism responsible for the switch to monoclonal proliferation of uremic parathyroid cells is not completely understood. A reduced number of calcium-sensing receptors and vitamin D receptors in areas of nodular transformation may contribute both to the progression of SHPT and to the abnormal proliferation of parathyroid cells, leading to the formation of a hyperfunctioning parathyroid nodule or adenoma in patients with ESRD [49].

This study had two main limitations. First, some other parameters that might be predictors of HBS, such as bone mineral density and bone histomorphometry, were not routinely measured. Second, we only have short-term follow-up data on our patients and thus cannot determine whether HBS affects future patient outcomes.

## Conclusions

This study demonstrated that HBS is common and associated with long hospital stays after parathyroidectomy in dialysis patients with SHPT. Young age (≤45 years), high preoperative serum ALP (>420 IU) and iPTH (>1000 pg/mL), and absence of preoperative hypercalcemia (>10.2 mg/dL) were independent risk factors of HBS. Early identification of HBS and initiation of oral calcium and active vitamin D analogues are suggested in post-parathyroidectomy patients at high risk of developing HBS.
